# Health-Related Quality of Life Impacts Mortality but Not Progression to End-Stage Renal Disease in Pre-Dialysis Chronic Kidney Disease: A Prospective Observational Study

**DOI:** 10.1371/journal.pone.0165675

**Published:** 2016-11-10

**Authors:** Mark D. Jesky, Mary Dutton, Indranil Dasgupta, Punit Yadav, Khai Ping Ng, Anthony Fenton, Derek Kyte, Charles J. Ferro, Melanie Calvert, Paul Cockwell, Stephanie J. Stringer

**Affiliations:** 1 Department of Renal Medicine, University Hospitals Birmingham NHS Foundation Trust, Birmingham, United Kingdom; 2 Institute of Translational Medicine, College of Medical and Dental Sciences, University of Birmingham, Birmingham, United Kingdom; 3 Renal Unit, Heart of England NHS Foundation Trust, Birmingham, United Kingdom; 4 Institute of Cardiovascular Sciences, College of Medical and Dental Sciences, University of Birmingham, Birmingham, United Kingdom; 5 Institute of Applied Health Research, College of Medical and Dental Sciences, University of Birmingham, Birmingham, United Kingdom; The University of Tokyo, JAPAN

## Abstract

**Background:**

Chronic kidney disease (CKD) is associated with reduced health-related quality of life (HRQL). However, the relationship between pre-dialysis CKD, HRQL and clinical outcomes, including mortality and progression to end-stage renal disease (ESRD) is unclear.

**Methods:**

All 745 participants recruited into the Renal Impairment In Secondary Care study to end March 2014 were included. Demographic, clinical and laboratory data were collected at baseline including an assessment of HRQL using the Euroqol EQ-5D-3L. Health states were converted into an EQ-5D_index_ score using a set of weighted preferences specific to the UK population. Multivariable Cox proportional hazards regression and competing risk analyses were undertaken to evaluate the association of HRQL with progression to ESRD or all-cause mortality. Regression analyses were then performed to identify variables associated with the significant HRQL components.

**Results:**

Median eGFR was 25.8 ml/min/1.73 m^2^ (IQR 19.6–33.7ml/min) and median ACR was 33 mg/mmol (IQR 6.6–130.3 mg/mmol). Five hundred and fifty five participants (75.7%) reported problems with one or more EQ-5D domains. When adjusted for age, gender, comorbidity, eGFR and ACR, both reported problems with self-care [hazard ratio 2.542, 95% confidence interval 1.222–5.286, p = 0.013] and reduced EQ-5D_index_ score [hazard ratio 0.283, 95% confidence interval 0.099–0.810, p = 0.019] were significantly associated with an increase in all-cause mortality. Similar findings were observed for competing risk analyses. Reduced HRQL was not a risk factor for progression to ESRD in multivariable analyses.

**Conclusions:**

Impaired HRQL is common in the pre-dialysis CKD population. Reduced HRQL, as demonstrated by problems with self-care or a lower EQ-5D_index_ score, is associated with a higher risk for death but not ESRD. Multiple factors influence these aspects of HRQL but renal function, as measured by eGFR and ACR, are not among them.

## Introduction

Chronic kidney disease (CKD) affects up to one in seven adults [1–3] and is associated with an increased risk of all-cause and cardiovascular mortality, and end-stage renal disease (ESRD) [4, 5]. There is increasing evidence of an association between pre-dialysis CKD and impaired health-related quality of life (HRQL) as assessed by a variety of patient reported outcome measures (PROMs) [6–9].

HRQL can be assessed using disease specific or generic instruments. The use of different PROMs within different populations to evaluate HRQL means that it is difficult to assess the relevance of the results reported. Furthermore, there are limited quantifiable data on the relationship between HRQL scores and clinical outcomes, including mortality and progression to ESRD. Previous studies have either been small, investigating these outcomes in a Taiwanese population [10], or have focused on individuals of black ethnicity with hypertensive CKD in the United States [11]. A recently published study investigated the impact of HRQL using a kidney disease specific tool (KDQOL-36) and found that low HRQL was independently association with CV events and death, but not CKD progression [12].

A systematic review of PROMs used in CKD supported the use of preference-based utility measures, favouring the EuroQol, EQ-5D due to ease of use for patients and for the ability to derive utility values for health economic evaluation [13].

To date, there have been few studies investigating the relationship between pre-dialysis CKD and HRQL as measured by EQ-5D [6–8], and no studies examining the relationship between EQ-5D scores and clinical outcomes. To address this we evaluated HRQL within a large prospective cohort study of pre-dialysis CKD, where EQ-5D was collected at recruitment, to assess the relationship between HRQL and CKD stage, and the impact of HRQL on risk of death or progression to ESRD.

## Materials and Methods

The Renal Impairment In Secondary Care (RIISC) study (NCT01722383) was approved by South Birmingham Research Ethics Committee (reference: 10/H1207/6). Patient recruitment commenced in October 2010, the methodology utilised has been described in detail elsewhere [14, 15].

In brief, RIISC is a two-centre, United Kingdom based, prospective observational cohort study with the aim of assessing determinants of long-term outcomes in patients with high risk CKD. Inclusion criteria comprised patients with pre-dialysis CKD who fulfilled criteria for secondary care follow-up as defined by the UK National Institute for Health and Care Excellence (NICE) 2008 CKD guidelines [16] (an MDRD eGFR below 30ml/min/1.73m^2^ or an eGFR 30-59ml/min/1.73m^2^ with either decline of ≥5 mls/min/1.73m^2^/year or ≥10 mls/min/1.73m^2^/5 years or an urinary albumin creatinine ratio (ACR) ≥70 mg/mmol on three occasions) and therefore are considered at high risk of progression to ESRD. Individuals requiring immunosuppression for immune-mediated renal disease, or who had commenced renal replacement therapy (RRT), were not eligible for recruitment All patients provided written consent and the study was conducted in accordance with the Declaration of Helsinki.

The patients consented for follow-up for ten years from recruitment. Blood and urine samples were collected and processed according to pre-defined standard operating procedures and stored at -80°C until analysis [14]. Patient mortality was captured through linkage between electronic patient records and the Office of National Statistics, which collects information on all registered deaths in the United Kingdom. Progression to ESRD was defined as the initiation of RRT (chronic dialysis or renal transplantation).

Reporting of the study conforms to the STROBE statement for reporting of observational studies [17, 18].

### Quality of life

Data were collected from participants using the EQ-5D-3L (abbreviated to EQ-5D throughout this manuscript). This is a validated, generic preference-based measure of health status that comprises a 5-question multi-attribute questionnaire and a visual analogue self-rating scale (VAS) [19]. Respondents were asked to rate severity of their current problems (level 1 = no problems, level 2 = some/moderate problems, level 3 = severe/extreme problems) for five dimensions of health: mobility, self-care, usual activities, pain/discomfort, and anxiety/depression). Health states were converted into an EQ-5D_index_ score ranging from −0.594 to 1.0 (where 1 is full health and lower values indicate worse HRQL) using a set of weighted preferences produced from the UK population [20]. The EQ VAS asks respondents to rate their own health state relative to full health (score = 100) or worst imaginable health state (score = 0).

### Socio-economic status

Socio-economic status (SES) was assessed using the Index of Multiple Deprivation (IMD 2010) [21]; an individual was assigned a score and rank according to their postcode; lower scores and ranks indicated greater deprivation. The IMD has been validated as superior to traditional deprivation indices such as the Townsend score due to its use of multiple domains reflective of socioeconomic deprivation [22].

Educational attainment was defined by established UK education milestones (no formal qualifications, GCSE/O’Level, NVQ, A’Level, undergraduate, post-graduate). Current employment status was subdivided into the following categories: in employment, unemployed or retired. Individuals were then asked to state the occupation category best describing their current or last employment.

### Demographic, Clinical and Laboratory Variables

Demographic factors included in the analysis comprised: age, gender, ethnicity, SES (the most deprived IMD quintile was compared to the other four quintiles combined), educational attainment, current employment status, smoking history, and alcohol consumption.

Clinical factors comprised comorbidity, presented by individual comorbidity (malignancy, diabetes mellitus, chronic obstructive airways disease, cerebrovascular disease, ischaemic heart disease and peripheral vascular disease) and Charlson Comorbidity Index (CCI) [23]. Other factors included body mass index (BMI), and blood pressure. Laboratory measurements comprised kidney function (MDRD eGFR corrected for ethnicity [24] and ACR), haemoglobin, acidaemia (serum bicarbonate), serum albumin, and C-reactive protein (CRP).

Brachial blood pressure (BP) was measured using the BpTRU fully automated and validated sphygmomanometer (BpTRU Medical Devices, Coquitlam, BC, Canada), which obtained a series of six BP readings at one-minute intervals following a five-minute rest period. Mean BP was derived from the average of the second to sixth BP reading. This average reading has been reported as comparable to mean daytime BP from 24 hour ambulatory BP monitoring [25].

Biochemistry results from the clinical laboratory were obtained from tests performed in accordance with the current standard of care. CRP was measured using the Full Range C-Reactive Protein Kit on a SPA^™^ automated PLUS turbidimeter (The Binding Site Group Ltd, Birmingham, UK).

### Statistical analyses

Analyses were performed using Stata 13.1 (Statacorp, College Station, Texas, USA).

#### Descriptive statistics

Descriptive statistics are presented as a complete cohort. Data are presented as mean with standard deviation (SD) or median with interquartile range (IQR) depending on distribution. Continuous variables were compared using ANOVA (parametric distribution) or Kruskal-Wallis (non-parametric distribution). Categorical variables were compared using chi-squared tests. Statistical significance was defined as a two-tailed p-value <0.05.

#### Survival Analyses

Cox proportional hazard analyses (Stata command stcox) were performed for end-points of death and ESRD (censor date March 2014). The proportionality assumption was assessed using log-log plots. Data are presented using hazard ratios (HR]) with 95% confidence intervals (CI), p-values and survival plots.

Individual constituents of the EQ-5D were analysed (univariable analyses). Any components demonstrating p<0.1 were then included in multivariable analyses together with *a priori* variables (age, gender, comorbidity assessed by CCI, eGFR and ACR).

#### Competing Risk Analyses

Survival analyses, by their nature, use time-to-event data [26]. In this study we investigated two events (end-points): death and progression to ESRD. Survival analyses including Cox proportional hazard analyses treat all censored events as ‘uninformative’; that is to say a patient being censored due to reaching the end of their follow up or due to another end-point (death in the case of ESRD or vice-versa) are treated equally. As these other events are of clinical significance and of statistical importance; someone who has died will never reach ESRD [27]. Therefore, in order to incorporate this into analyses, we carried out competing risk analyses according to the method described by Fine and Gray [28] (Stata command stcrreg).

#### Regression analyses to assess the impact of demographic, clinical and laboratory variables on HRQL

Logistic regression was performed to analyse the relationship between problems in each of the five domains with clinical, demographic and laboratory variables using dichotomised data (patients with moderate and severe problems in a domain were combined and compared to those with no problems). Odds ratios with 95% CI and two-tailed p-values are presented.

Linear regression was utilised for the calculated EQ-5D_index_ score and the EQ VAS (coefficient with 95% CI and p-value). Residual plots were evaluated to determine appropriateness of linear regression models.

Data were entered into multivariable analyses if p<0.1 and a backwards selection model performed until remaining variables had a p<0.05. Goodness-of-fit is indicated by pseudo R^2^ (logistic regression) or R^2^ (linear regression) values.

## Results

### Descriptive Statistics

**Fig 1:**
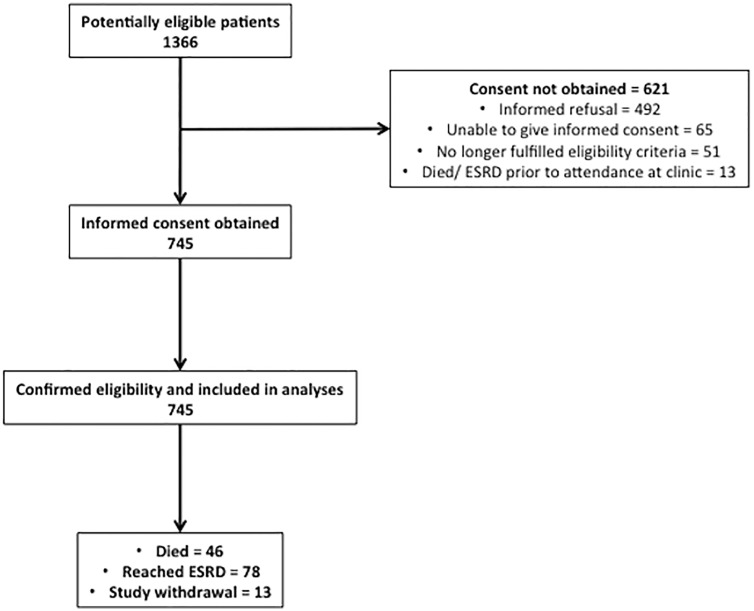
Flow diagram of the participants in the study. ESRD—End-stage renal disease.

All participants recruited to end March 2014 (n = 745) were included in the study. Fig 1 indicates the number of individuals at each stage of evaluation. Baseline demographic, clinical and laboratory data are shown in Table 1 (see S1 Table for data split by CKD stage). Median age at recruitment was 64 years (IQR 50–76 years) and 60.8% were male. The proportion of male participants decreased with lower CKD stage (p = 0.045). 68.1% were of white ethnicity, 20.1% south-Asian, 9.4% black ethnicity, and 2.4% from other ethnic groups. There was a borderline difference in ethnicity by CKD stage (p = 0.052).

**Table 1:** Demographic, clinical and laboratory data. Categorical variables are expressed as number (%), and continuous variables as mean (SD) or median (IQR).

	Cohort (n = 745)	Data completeness (%)
**DEMOGRAPHIC FACTORS**		
**Age (years)**	64 (50–76)	100
**Gender—Female (%)**	39.2	100
**Ethnicity**		
** White (%)**	68.1	100
** South Asian (%)**	20.1	
** Black (%)**	9.4	
** Other (%)**	2.4	
**SES (IMD 2010)**		
** Score**	31.9 (46.7–35.1)	99.2
** % in most deprived national quintile**	46.3	99.5
**Educational Attainment**		
** No formal qualifications (%)**	46.5	**100**
** GCSE/ O'level (%)**	21.8	
** NVQ (%)**	9.1	
** A'Level (%)**	7.5	
** Undergraduate (%)**	10.0	
** Postgraduate (%)**	5.1	
**Current Employment Status**		
** Not currently in employment (%)**	19.1	**100**
** In Employment (%)**	28.5	
** Retired (%)**	52.5	
**Job Type (when last working)**		
** None (%)**	0.2	**100**
** Unskilled manual (%)**	21.4	
** Skilled manual (%)**	38.2	
** Clerical (%)**	12.9	
** Managerial (%)**	10.0	
** Professional (%)**	17.4	
**Smoking Status**		
** Never (%)**	48.0	99.3
** Current (%)**	12.8	
** Previous (%)**	39.2	
**Alcohol Consumption**		
** None (%)**	57.7	100
** 1–10 unit (%)**	29.7	
** 11–20 units (%)**	8.6	
** >20 units (%)**	4.0	
**BMI (kg/m2)**	28.6 (24.9–33.21)	98.5
**CLINICAL FACTORS**		
**Individual Comorbidities**		
** Malignancy (%)**	14.0	100
** Diabetes (%)**	34.0	
** COPD (%)**	10.0	
** Cerebrovascular disease (%)**	11.3	
** IHD (%)**	21.2	
** PVD (%)**	9.5	
**Comorbidity Scores**		
** CCI**	3 (1–5)	99.9
** Age Adjusted CCI**	5 (2–8)	99.9
**Blood Pressure**		
** Systolic BP (mmHg)**	130.5 (20.5)	98.7
** Diastolic BP (mmHg)**	76.5 (12.6)	98.7
**BIOLOGICAL MARKERS**		
**Creatinine (μmol/L)**	212 (166.5–271.5)	98.4
**eGFR (ml/min/1.73m2)**	25.8 (19.6–33.7)	98.4
**ACR (mg/mmol)**	33 (6.6–130.3)	94.5
**Haemoglobin (g/L)**	124.3 (17.2)	95.6
**Bicarbonate (mmol/L)**	24.0 (3.6)	96.8
**Albumin (g/L)**	43 (43–46)	98
**CRP (mg/L)**	3.0 (1.4–7.2)	93.8

SES—Socio-economic status

BMI—Body Mass Index

CCI—Charlson Comorbidity index

BP—Blood Pressure

eGFR—estimated Glomerular Filtration Rate

ACR—Albumin Creatinine Ratio

CRP—C-reactive protein

46.3% of participants were in the most deprived quintile nationally (IMD 2010). No difference in SES was seen when analysed by CKD stage for IMD score (p = 0.517) or comparing the percentage in the most deprived quintile (p = 0.351). Comorbidity was common and increased with advancing CKD stage, both as assessed by individual comorbidities and the CCI (p = 0.007; age adjusted CCI p<0.001).

Table 2 illustrates the study population by Kidney Disease Improving Global Outcomes (KDIGO) classification [29]. Median eGFR was 25.8 ml/min/1.73 m^2^ (IQR 19.6–33.7ml/min) and Median ACR was 33 mg/mmol (IQR 6.6–130.3 mg/mmol).

**Table 2:** Study population by Kidney Disease Improving Global Outcomes (KDIGO) classification [29].

			ACR (mg/mmol)			
			<3	3–30	>30	ACR not
			A1	A2	A3	stated
**MDRD eGFR (ml/min/1.73m2)**	**≥60**	**G1/G2**	1	1	25	2
	**45–59**	**G3a**	8	13	24	0
	**30–44**	**G3b**	28	59	78	8
	**15–29**	**G4**	66	138	192	27
	**<15**	**G5**	5	19	47	4

### Self-Reported HRQL

**Fig 2:**
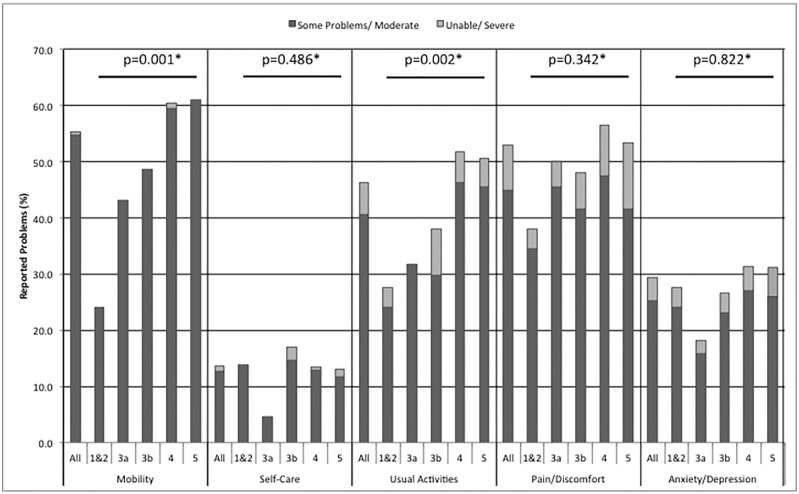
Reported HRQL Problems by EQ-5D domain. Data presented as whole cohort (All) and catagorised by CKD stage (determined by MDRD eGFR). * p-value for chi-squared test comparing CKD stage to reported problems for each EQ-5D-3L domain.

Complete HRQL data were available for 733 participants (98.4%). Proportions of individuals reporting problems with each of the five domains are shown in Fig 2. One hundred and seventy eight participants (24.3%) reported no problems within any domain. Problems with one, two, three, four and five domains were reported by 136 (18.6%), 129 (17.6%), 153 (20.9%), 91 (12.4%) and 46 (6.3%) participants, respectively.

### Associations between HRQL and CKD

As illustrated in Fig 2, statistically significant differences between CKD stages were seen in the mobility (p = 0.001) and usual activity (p = 0.002) domains, with more problems reported with a worse CKD stage. No significant difference was found between CKD stages and the other domains.

Only a small number of participants described problems in the unable/severe category, therefore data were dichotomised to combine the respondents who reported moderate problems with those in the severe or unable category.

Health related quality of life for the EQ VAS and calculated EQ-5D_index_ score are shown in Table 3. The EQ-5D_index_ score decreased (worsened) with more advanced CKD stage (p = 0.017). No significant difference was seen between CKD stage and the EQ VAS.

**Table 3:** Calculated EQ-5D Index Score and Visual Analogue Scale by CKD stage.

	EQ-5D Index Score	Visual Analogue Scale
**All**	0.74 (0.66–0.88)	65 (50–80)
**Stage G1/G2**	0.85 (0.70–1)	50 (75–82.5)
**Stage G3a**	0.80 (0.69–1)	70 (50–80)
**Stage G3b**	0.80 (0.68–1)	70 (50–80)
**Stage G4**	0.74 (0.62–0.85)	60 (50–80)
**Stage G5**	0.73 (0.62–1)	55 (50–80)
**p-value**	0.017	0.094

### Association between HRQL and Clinical end-points

#### Death

**Fig 3:**
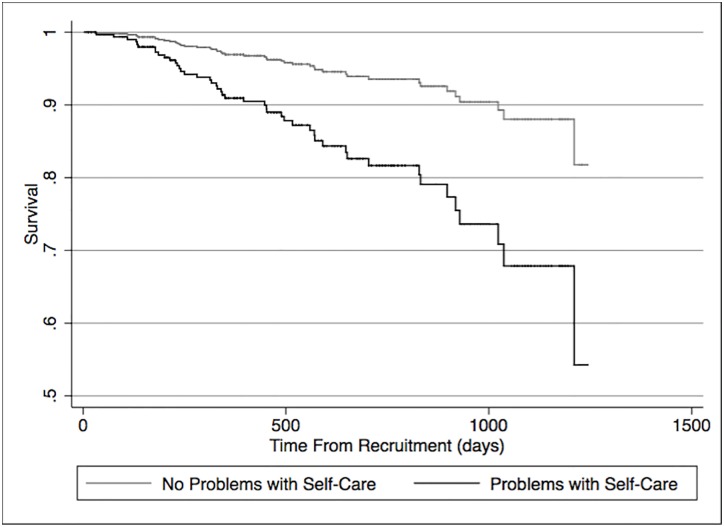
Cox Proportional Hazards Regression for reported problems with self-care and death. Univariable Analyses.

By March 24^th^ 2014, 46 (6.2%) participants had died. Univariable cox regression analysis demonstrated that reported problems with mobility, self-care (Fig 3), usual, lower EQ-5D_index_ score, and lower EQ VAS, were all associated with an increased risk of death. Table 4 indicates univariable cox regression analyses for a priori variables and EQ-5D components.

**Table 4:** Univariable Survival Analyses (Cox regression) for hazard ratio (HR) for death. A priori variables and all components of EQ-5D shown.

		95% CI		
	HR	Lower	Higher	P-Value
**A Priori Variables**				
** Age (per 10 year increase)**	2.420	1.788	3.276	<0.001
** Gender (female as reference)**	1.375	0.742	2.550	0.311
** Charlson Comorbidity Index**	1.408	1.254	1.581	<0.001
** eGFR (per 5ml/min increase)**	0.814	0.689	0.961	0.015
** ACR (per 10mg/mmol rise)**	1.016	0.999	1.034	0.065
**EQ-5D Components**				
** Mobility**	3.725	1.795	7.728	<0.001
** Self-Care**	3.042	1.620	5.710	0.001
** Usual Activities**	3.024	1.591	5.748	0.001
** Pain/Discomfort**	1.109	0.621	1.982	0.727
** Anxiety/ Depression**	1.559	0.856	2.838	0.146
** EQ-5D Index Score**	0.200	0.088	0.454	<0.001
** Visual Analogue Scale**	0.976	0.963	0.989	<0.001

**Fig 4:**
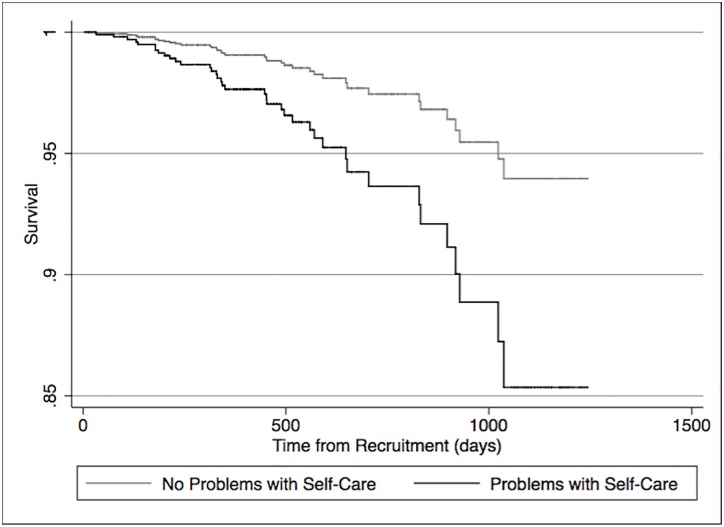
Cox Proportional Hazards Regression for reported problems with self-care and death. Multivariable Analyses. Covariates included age, gender, Comorbidity (assessed by Charlson Comorbidity Index) and renal function (eGFR and ACR).

In multivariable analysis, each significant EQ-5D variable was combined with age, gender, comorbidity assessed by CCI, eGFR and ACR. Self-care (HR 2.542, 95% CI 1.222–5.286, p = 0.013, Fig 4, Table 5) and the EQ-5D_index_ score (HR 0.283, 95% CI 0.099–0.810, p = 0.019, Table 5) were independently associated with an increased risk of death. Fourteen out of 102 (13.7%) participants who reported problems with self-care died compared to 32/641 (5.0%) participants who reported no problems (chi-squared p = 0.001).

**Table 5:** Multivariable Survival Analyses (Cox regression and Competing risk) for hazard ratio (HR) and subdistribution hazard ratio (SHR) for death.

	Cox Regression Analyses				Competing Risk Analyses			
		95% CI				95% CI		
	HR	Lower	Higher	P-Value	SHR	Lower	Higher	P-Value
**Identified problem with self care**	2.542	1.222	5.286	0.013	2.608	1.260	5.397	0.01
**Age (per 10 year increase)**	2.040	1.444	2.883	<0.001	2.243	1.592	3.161	<0.001
**Gender (female as reference)**	1.500	0.684	3.290	0.311	1.442	0.678	3.067	0.341
**Charlson Comorbidity Index**	1.239	1.064	1.443	0.006	1.194	1.044	1.366	0.01
**eGFR (per 5ml/min increase)**	0.854	0.696	1.047	0.128	0.905	0.747	1.096	0.306
**ACR (per 10mg/mmol rise)**	1.019	1.004	1.035	0.013	1.016	1.000	1.032	0.045
**EQ-5D index score**	0.283	0.099	0.810	0.019	0.317	0.105	0.958	0.042
**Age (per 10 year increase)**	2.093	1.482	2.954	<0.001	2.308	1.656	3.215	<0.001
**Gender (female as reference)**	1.570	0.731	3.373	0.247	1.430	0.702	2.911	0.324
**Charlson Comorbidity Index**	1.202	1.029	1.404	0.02	1.155	1.013	1.317	0.032
**eGFR (per 5ml/min increase)**	0.851	0.692	1.046	0.126	0.909	0.747	1.107	0.344
**ACR (per 10mg/mmol rise)**	1.024	1.009	1.040	0.002	1.019	1.003	1.035	0.016

To adjust the HR associated for death for the competing end-point of ESRD, a competing risk analysis was performed. Problems with self-care (sub-distribution hazard ratio [SHR] 2.608, 95% CI 1.260–5.597, p = 0.01) and a lower EQ-5D_index_ score (SHR 0.317, 95% CI 0.105–0.958, p = 0.042) remained significant in the multivariable analysis with age, gender, comorbidity, eGFR and ACR (Table 5).

These analyses also identify increasing age, comorbidity and higher ACR as being associated with death. Estimated GFR was not significant; however a creatinine greater than 265 μmol/L (3mg/dL) *scores* 2 points in the CCI. Reanalysing the data for the CCI *without* the renal disease points results in eGFR demonstrating significance in Cox regression but not competing risk analyses (see S2 Table).

#### End-Stage Renal Disease

Seventy-eight participants (10.5%) had reached ESRD by the censor date. Lower EQ VAS score was the only component of the EQ-5D associated with an increased HR for progression to ESRD (Table 6). Significance was lost when age, gender, comorbidity, eGFR and ACR were included in a multivariable analysis. Similarly, competing risk analysis indicated an association with a lower VAS and ESRD in univariable but not multivariable analysis.

**Table 6:** Univariable Survival Analyses (Cox regression) for hazard ratio (HR) for End-stage Renal Disease (ESRD). A priori variables and all components of EQ-5D shown.

		95% CI		
	HR	Lower	Higher	P-Value
**A Priori Variables**				
** Age (per 10 year increase)**	0.843	0.739	0.961	0.011
** Gender (female as reference)**	1.013	0.644	1.595	0.955
** Charlson Comorbidity Index**	1.195	1.091	1.309	<0.001
** eGFR (per 5ml/min increase)**	0.406	0.337	0.490	<0.001
** ACR (per 10mg/mmol rise)**	1.034	1.025	1.043	<0.001
**EQ-5D Components**				
** Mobility**	1.001	0.641	1.562	0.998
** Self-Care**	0.587	0.255	1.352	0.211
** Usual Activities**	1.255	0.805	1.956	0.317
** Pain/Discomfort**	1.093	0.700	1.707	0.695
** Anxiety/ Depression**	1.171	0.724	1.895	0.519
** EQ-5D Index Score**	1.023	0.444	2.354	0.958
** Visual Analogue Scale**	0.988	0.977	0.998	0.016

### The impact of demographic, clinical and laboratory variables on HRQL

The analyses above demonstrate the two HRQL factors associated with death in the survival analyses were problems with self-care and a lower EQ-5D_index_ score. In order to explore factors predictive of these two elements, further exploratory analyses were performed for self-care (logistic regression) and the EQ-5D_index_ score (linear regression).

#### Self-care

Table 7 shows the factors that were associated with (p<0.1) reported problems with self-care.

**Table 7:** Variables predictive of reported problems with self-care by logistic regression.

	Univariable Analyses					Multivariable Analyses*				
	OR	Confidence interval		SE	p	OR	Confidence interval		SE	p
		Lower	Upper				Lower	Upper		
**Age (per 10 year increase)**	1.290	1.118	1.487	0.094	<0.001					
**Ethnicity (white as reference)**										
** South Asian**	1.456	0.878	2.415	0.376	0.146	2.058	1.115	3.798	0.643	0.021
** Black**	1.357	0.676	2.725	0.483	0.391	1.051	0.420	2.632	0.492	0.914
** Other/ Not stated**	2.799	0.965	8.125	1.522	0.058	4.277	1.247	14.668	2.689	0.021
**SES (most deprived Quintile)**	1.506	0.987	2.299	0.325	0.058					
**Academic Qualifications (none versus some)**	2.157	1.402	3.319	0.474	<0.001					
**Employment status (currently employed as reference)**										
** Not employed**	26.018	6.087	111.212	19.283	<0.001	15.675	3.536	69.477	11.908	<0.001
** Retired**	23.774	5.771	97.941	17.173	<0.001	19.460	4.651	81.413	14.210	<0.001
**Weekly Alcohol Consumption (none as reference)**										
** Under 10 units**	0.543	0.323	0.911	0.143	0.021					
** 11–10 units**	0.970	0.471	1.999	0.358	0.935					
** More than 20 units**	0.177	0.024	1.323	0.182	0.092					
**BMI (kg/m2)**	1.074	1.043	1.106	0.016	<0.001	1.058	1.023	1.095	0.019	0.001
**CCI**	1.209	1.111	1.315	0.052	<0.001					
**DM**	2.412	1.580	3.682	0.521	<0.001					
**COPD**	2.606	1.503	4.520	0.732	0.001					
**IHD**	1.793	1.136	2.829	0.417	0.012					
**PVD**	2.154	1.211	3.832	0.633	0.009					
**SBP (mmHg)**	1.010	1.000	1.020	0.005	0.06					
**DBP (mmHg)**	0.980	0.963	0.997	0.009	0.019					
**Haemoglobin (g/L)**	0.984	0.972	0.997	0.006	0.015					
**Bicarbonate (mmol/L)**	1.101	1.035	1.171	0.034	0.002	1.102	1.023	1.095	0.039	0.007
**Albumin (g/L)**	0.949	0.910	0.989	0.020	0.012					
**CRP (mg/L)**	1.017	1.004	1.030	0.007	0.009					
**log CRP**	1.541	1.288	1.844	0.141	<0.001	1.282	1.041	1.580	0.137	0.020

* Significant variables removed in a backwards stepwise technique until remaining variables had a p<0.05.

SES—Socio-economic Status

BMI—Body mass index

CCI—Charlson Comorbidity Index

DM—Diabetes mellitus

COPD—Chronic Obstructive Airways Disease

IHD—Ischaemic Heart Disease

PVD—Peripheral vascular disease

SBP—Systolic Blood Pressure

DBP—Diastolic Blood Pressure

CRP—C-reactive protein

Ethnicity classified as other or not stated, people who were not currently working, higher BMI, higher bicarbonate concentration, and higher CRP were statistically significantly associated with reported problems with self-care in multivariable analysis (Table 7). This model explained 16.5% of variability with self-care (pseudo R^2^ 0.165). Of note, age and renal function did not influence this aspect of HRQL.

#### EQ-5D_index_ score

Table 8 shows factors (p<0.1) associated with a higher EQ-5D score (i.e. better HRQL).

**Table 8:** Variables predictive of higher EQ-5D index score by linear regression.

	Univariable Analyses					Multivariable Analyses*				
	Coefficient	Confidence interval		SE	p	Coefficient	Confidence interval		SE	p
		Lower	Upper				Lower	Upper		
**Age (per 10 year increase)**	-0.034	-0.046	-0.022	0.006	<0.001					
**Gender (male as reference)**	0.042	0.000	0.084	0.021	0.048	0.045	0.005	0.086	0.021	0.029
**SES (most deprived Quintile)**	-0.060	-0.101	-0.019	0.021	0.004					
**Academic Qualifications (none versus some)**	-0.010	-0.137	-0.056	0.021	<0.001					
**Employment status (currently employed as reference)**										
** Not employed**	-0.263	-0.320	-0.207	0.029	<0.001	-0.199	-0.255	-0.143	0.033	<0.001
** Retired**	-0.206	-0.251	-0.162	0.023	<0.001	-0.122	-0.173	-0.072	0.026	<0.001
**Smoking status (non smoker as reference)**										
** Current**	-0.068	-0.133	-0.004	0.033	0.037	-0.104	-0.164	-0.044	0.031	0.001
** Previous**	-0.067	-0.111	-0.023	0.022	0.003	-0.028	-0.070	0.015	0.022	0.205
**Weekly Alcohol Consumption (none as reference)**										
** Under 10 units**	0.073	0.027	0.119	0.023	0.002					
** 11–10 units**	0.043	-0.032	0.117	-0.038	0.261					
** More than 20 units**	0.173	0.070	0.277	0.053	0.001					
**BMI (kg/m2)**	-0.009	-0.012	-0.006	0.002	<0.001	-0.006	-0.009	-0.003	0.002	<0.001
**CCI**	-0.028	-0.036	-0.019	0.004	<0.001	-0.014	-0.024	-0.005	0.005	0.003
**DM**	-0.108	-0.149	-0.066	0.021	<0.001	-0.449	-0.087	-0.003	0.022	0.037
**COPD**	-0.102	-0.168	-0.035	0.034	0.001					
**CVD**	-0.094	-0.156	-0.032	0.032	0.003					
**IHD**	-0.127	-0.175	-0.079	0.025	<0.001	-0.056	-0.104	-0.008	0.024	0.021
**PVD**	-0.143	-0.210	-0.075	0.420	<0.001					
**SBP (mmHg)**	-0.002	-0.003	-0.001	0.001	0.001					
**DBP (mmHg)**	0.002	0.000	0.004	0.001	0.011					
**eGFR (per 5ml/min)**	0.009	0.003	0.015	0.003	0.006					
**Haemoglobin (g/L)**	0.002	0.001	0.004	0.001	<0.001					
**Albumin (g/L)**	0.007	0.003	0.012	0.002	0.001					
**CRP (mg/L)**	-.0.003	-0.005	-0.001	0.001	<0.001					
**log CRP**	-0.051	-0.068	-0.033	0.008	<0.001	-0.021	-0.038	-0.4	0.008	0.013

* Significant variables removed in a backwards stepwise technique until remaining variables had a p<0.05.

SES—Socio-economic Status

BMI—Body mass index

CCI—Charlson Comorbidity Index

DM—Diabetes mellitus

COPD—Chronic Obstructive Airways Disease

IHD—Ischaemic Heart Disease

PVD—Peripheral vascular disease

SBP—Systolic Blood Pressure

DBP—Diastolic Blood Pressure

CRP—C-reactive protein

Multivariable analysis found the following variables remained associated with better HRQL: male gender; currently in employment; not smoking in comparison to current smoking; lower BMI; less comorbidity; and lower CRP (Table 8). This linear regression model explained 20.8% of the variability in HRQL as assessed by the EQ-5D_index_ score (adjusted R^2^ 0.208). Again age and renal function were not associated with this assessment of overall HRQL.

## Discussion

The relationship between pre-dialysis CKD, HRQL and clinical outcomes is an important aspect of nephrology practice. Our study, conducted in a cohort of people with advanced and/or progressive CKD, demonstrated that reported problems with HRQL, as measured by the EQ-5D, were common; only 24.3% of participants reported no problem in any EQ-5D domain.

Impaired HRQL was a risk factor for death; problems with self-care and overall HRQL, assessed by the EQ-5D_index_ score, were associated with an increased HR for death when analysed with age, gender, comorbidity, eGFR and ACR. This association was present in both cox proportional hazard regression and competing risk regression (with ESRD as the competing risk). No element of HRQL was independently associated with risk of progression to ESRD.

Until recently, previous studies investigating HRQL in patients with pre-dialysis CKD had focused on specific populations (Taiwanese [10] or individuals of black ethnicity with hypertensive CKD in the United States [11]). Whilst the generalizability of these studies to a multi-ethnic United Kingdom CKD population is questionable, both studies identified an association with HRQL and death in similarity to our study. However, the association with HRQL and CKD progression was conflicting; Tsai and colleagues identified an association [10] but Porter and colleagues only noted an association in a composite of death and CKD progression [11].

A combined analysis of the Chronic Renal Insufficiency Cohort and Hispanic Chronic Renal Insufficiency Cohort has recently been published [12]. 3837 patients (of a total of 3939 enrolled) completed the disease specific KDQOL-36 questionnaire. Consistent with our study, they found that low HRQL was independently associated with a higher risk of death but not CKD progression in several KDQOL-36 subscales (physical component summary, mental component subscale, effects and symptoms). The KDQOL-36 questionnaire is a detailed HRQL survey based on a chronic disease core, with added items relevant to patients with kidney disease [30]. Compared to the EQ-5D, it is more time consuming to complete, has some components that apply more to those undergoing RRT than the pre-dialysis population, and its utility in health economic evaluations is not as established.

To explore further the factors that influence the components of HRQL associated with death (self-care and the EQ-5D_index_ score), we assessed the relationship between these components and demographic, clinical and laboratory variables utilizing regression analysis. Not being currently employed, whether young and not working or retired, conveyed the highest HR associated with impaired HRQL. Other significant factors for a lower HRQL included higher BMI, a higher CRP, and multimorbidity. Further research is warranted.

Interestingly, we did not identify an association between these aspects of HRQL and SES, increasing age of the participants or renal function, as measured by eGFR or ACR. This lack of association between HRQL and renal function, is a finding variably supported by previous studies [7, 31, 32].

As we, and others, have demonstrated, reported problems with HRQL are common in this population [13] and we have found an association between impaired HRQL and death. It is therefore important to consider what strategies could be used to improve HRQL; improving HRQL will not only improve patient well-being but may convey a survival advantage. Previous studies have demonstrated that optimisation of haemoglobin, psychological interventions and physical exercise may be of benefit [33–35]. However, the majority of these studies have focused on patients who have reached ESRD rather than pre-dialysis CKD. Therefore, the transferability of these, or of interventions instigated for other chronic disease states, requires evaluation.

In this study of patients with pre-dialysis CKD, problems with self-care and the EQ-5D_index_ score [20] were both of prognostic significance; in clinical practice problems with self-care may be the more useful HRQL screening question to identify patients with CKD at an increased risk of death. This could then direct social care resources towards these patients and ensure that appropriate time is allocated so that patients are adequately supported when counselled about their higher mortality risk. It may also enable identification of a high-risk group where interventions to improve outcomes can be studied.

### Strengths and Weaknesses

The major strength of this study is the use of a prospectively recruited, socio-economically and ethnically diverse cohort of patients with advanced and/or progressive pre-dialysis dependent CKD. Detailed demographic and clinical data were collected at initial recruitment and the participants were tracked longitudinally to record outcomes, including death and ESRD. HRQL was assessed by the EQ-5D tool, which is recommended as the preference based measure for HRQL evaluation in CKD [13]. Survival analyses were performed using both Cox proportional hazard analyses and competing risk analyses. The latter is important, though rarely used, as it enabled the competing risk of ESRD to be taken into account when assessing death and vice versa: both end-points are (separately) of key interest to patients, their families and clinicians [26, 36].

A weakness, as with all observational studies, is that we have assessed association rather than causation. Whilst the analyses for factors associated with an increased risk of death included baseline renal function and progression to ESRD (in competing risk analyses), we did not include any other measure of CKD progression. In addition, whilst we collected considerable demographic and clinical information, we did not complete any formal assessment of frailty, depression or nutritional status of the participants. These factors have been associated with impairment of HRQL [9, 37–42].

### Summary

In summary, we have demonstrated that impaired HRQL is common in a diverse pre-dialysis CKD population and that impaired HRQL, as demonstrated by problems with self-care or a lower EQ-5D_index_ score, is associated with a higher risk for death but not ESRD. Multiple factors influence these aspects of impaired HRQL but renal function, as measured by eGFR and ACR, are not among them.

Further studies are recommended to evaluate interventions that may improve HRQL within the pre-dialysis CKD population and to investigate whether any improvements in HRQL are associated with a survival advantage.

## Supporting Information

S1 Table Demographic, clinical and laboratory data subdivided by Kidney Disease Improving Global Outcomes (KDIGO) eGFR classification. (XLSX)

S2 Table Multivariable Survival Analyses for death. (XLSX)

## References

[1] Jain P, Calvert M, Cockwell P, McManus RJ. The need for improved identification and accurate classification of stages 3–5 Chronic Kidney Disease in primary care: retrospective cohort study. PLoS One. 2014;9(8):e100831 Epub 2014/08/15. 10.1371/journal.pone.0100831 25115813 PMC4130474

[2] Aitken GR, Roderick PJ, Fraser S, Mindell JS, O'Donoghue D, Day J, et al Change in prevalence of chronic kidney disease in England over time: comparison of nationally representative cross-sectional surveys from 2003 to 2010. BMJ Open. 2014;4(9):e005480 Epub 2014/10/02. 10.1136/bmjopen-2014-005480 25270853 PMC4179568

[3] Bruck K, Stel VS, Gambaro G, Hallan S, Volzke H, Arnlov J, et al CKD Prevalence Varies across the European General Population. J Am Soc Nephrol. 2015 Epub 2015/12/25. 10.1681/ASN.2015050542 . 26701975 PMC4926978

[4] Go AS, Chertow GM, Fan D, McCulloch CE, Hsu CY. Chronic kidney disease and the risks of death, cardiovascular events, and hospitalization. N Engl J Med. 2004;351(13):1296–305. Epub 2004/09/24. 10.1056/NEJMoa041031 . 15385656

[5] Astor BC, Matsushita K, Gansevoort RT, van der Velde M, Woodward M, Levey AS, et al Lower estimated glomerular filtration rate and higher albuminuria are associated with mortality and end-stage renal disease. A collaborative meta-analysis of kidney disease population cohorts. Kidney Int. 2011;79(12):1331–40. Epub 2011/02/04. 10.1038/ki.2010.550 . 21289598 PMC3917543

[6] Cohen SD, Patel SS, Khetpal P, Peterson RA, Kimmel PL. Pain, sleep disturbance, and quality of life in patients with chronic kidney disease. Clin J Am Soc Nephrol. 2007;2(5):919–25. Epub 2007/08/19. 10.2215/CJN.00820207 . 17702733

[7] Pagels AA, Soderkvist BK, Medin C, Hylander B, Heiwe S. Health-related quality of life in different stages of chronic kidney disease and at initiation of dialysis treatment. Health and quality of life outcomes. 2012;10:71 Epub 2012/06/20. 10.1186/1477-7525-10-71 22710013 PMC3511211

[8] Gorodetskaya I, Zenios S, McCulloch CE, Bostrom A, Hsu CY, Bindman AB, et al Health-related quality of life and estimates of utility in chronic kidney disease. Kidney Int. 2005;68(6):2801–8. Epub 2005/12/01. 10.1111/j.1523-1755.2005.00752.x . 16316356

[9] Abdel-Kader K, Unruh ML, Weisbord SD. Symptom burden, depression, and quality of life in chronic and end-stage kidney disease. Clin J Am Soc Nephrol. 2009;4(6):1057–64. Epub 2009/05/09. 10.2215/CJN.00430109 19423570 PMC2689883

[10] Tsai YC, Hung CC, Hwang SJ, Wang SL, Hsiao SM, Lin MY, et al Quality of life predicts risks of end-stage renal disease and mortality in patients with chronic kidney disease. Nephrol Dial Transplant. 2010;25(5):1621–6. Epub 2009/12/29. 10.1093/ndt/gfp671 . 20037172

[11] Porter A, Fischer MJ, Wang X, Brooks D, Bruce M, Charleston J, et al Quality of life and outcomes in African Americans with CKD. J Am Soc Nephrol. 2014;25(8):1849–55. Epub 2014/04/05. 10.1681/ASN.2013080835 24700865 PMC4116063

[12] Porter AC, Lash JP, Xie D, Pan Q, DeLuca J, Kanthety R, et al Predictors and Outcomes of Health-Related Quality of Life in Adults with CKD. Clin J Am Soc Nephrol. 2016;11(7):1154–62. Epub 2016/06/02. 10.2215/CJN.09990915 27246012 PMC4934840

[13] Gibbons E, Fitzpatrick R. A structured review of Patient-Reported Outcome Measures for people with Chronic Kidney Disease, 2010. University of Oxford, Group PROM; 2010.

[14] Stringer S, Sharma P, Dutton M, Jesky M, Ng K, Kaur O, et al The natural history of, and risk factors for, progressive chronic kidney disease (CKD): the Renal Impairment in Secondary care (RIISC) study; rationale and protocol. BMC Nephrol. 2013;14:95 Epub 2013/04/27. 10.1186/1471-2369-14-95 23617441 PMC3664075

[15] Sharma P, Dietrich T, Sidhu A, Vithlani V, Rahman M, Stringer S, et al The periodontal health component of the Renal Impairment In Secondary Care (RIISC) cohort study: a description of the rationale, methodology and initial baseline results. J Clin Periodontol. 2014;41(7):653–61. Epub 2014/04/18. 10.1111/jcpe.12263 . 24738870

[16] National Institute for Health and Clinical Excellence: CG73: Chronic Kidney Disease 2008. Available from: http://www.nice.org.uk/CG73.

[17] von Elm E, Altman DG, Egger M, Pocock SJ, Gotzsche PC, Vandenbroucke JP. The Strengthening the Reporting of Observational Studies in Epidemiology (STROBE) statement: guidelines for reporting observational studies. Lancet. 2007;370(9596):1453–7. Epub 2007/12/08. 10.1016/S0140-6736(07)61602-X . 18064739

[18] Simera I, Moher D, Hoey J, Schulz KF, Altman DG. A catalogue of reporting guidelines for health research. European journal of clinical investigation. 2010;40(1):35–53. Epub 2010/01/09. 10.1111/j.1365-2362.2009.02234.x . 20055895

[19] EuroQol—a new facility for the measurement of health-related quality of life. Health Policy. 1990;16(3):199–208. Epub 1990/11/05. . 10109801 10.1016/0168-8510(90)90421-9

[20] EQ-5D Value Sets: Inventory, Compratice review and User Guide. Netherlands: Springer; 2007.

[21] The English Indices of Deprivation 2010: Department for Communities and Local Government; 2011 [cited 2014 April 1]. Available from: https://www.gov.uk/government/uploads/system/uploads/attachment_data/file/6871/1871208.pdf.

[22] Jordan H, Roderick P, Martin D. The Index of Multiple Deprivation 2000 and accessibility effects on health. J Epidemiol Community Health. 2004;58(3):250–7. Epub 2004/02/18. 10.1136/jech.2003.013011 14966241 PMC1732697

[23] Charlson ME, Pompei P, Ales KL, MacKenzie CR. A new method of classifying prognostic comorbidity in longitudinal studies: development and validation. J Chronic Dis. 1987;40(5):373–83. Epub 1987/01/01. . 3558716 10.1016/0021-9681(87)90171-8

[24] Levey AS, Bosch JP, Lewis JB, Greene T, Rogers N, Roth D. A more accurate method to estimate glomerular filtration rate from serum creatinine: a new prediction equation. Modification of Diet in Renal Disease Study Group. Ann Intern Med. 1999;130(6):461–70. Epub 1999/03/13. . 10075613 10.7326/0003-4819-130-6-199903160-00002

[25] Brothwell S, Dutton M, Ferro C, Stringer S, Cockwell P. Optimising the accuracy of blood pressure monitoring in chronic kidney disease: the utility of BpTRU. BMC Nephrol. 2013;14:218 Epub 2013/10/12. 10.1186/1471-2369-14-218 24112304 PMC3852944

[26] Satagopan JM, Ben-Porat L, Berwick M, Robson M, Kutler D, Auerbach AD. A note on competing risks in survival data analysis. Br J Cancer. 2004;91(7):1229–35. Epub 2004/08/12. 10.1038/sj.bjc.6602102 15305188 PMC2410013

[27] Barbour SJ, Er L, Djurdjev O, Karim M, Levin A. Differences in progression of CKD and mortality amongst Caucasian, Oriental Asian and South Asian CKD patients. Nephrol Dial Transplant. 2010;25(11):3663–72. Epub 2010/04/07. 10.1093/ndt/gfq189 . 20368302

[28] Fine JP, Gray RJ. A proportional hazards model for the subdistribution of a competing risk. Journal of the American Statistical Association. 1999;94:496–509.

[29] Stevens PE, Levin A. Evaluation and management of chronic kidney disease: synopsis of the kidney disease: improving global outcomes 2012 clinical practice guideline. Ann Intern Med. 2013;158(11):825–30. Epub 2013/06/05. 10.7326/0003-4819-158-11-201306040-00007 . 23732715

[30] Hays RD, Kallich JD, Mapes DL, Coons SJ, Carter WB. Development of the kidney disease quality of life (KDQOL) instrument. Quality of life research: an international journal of quality of life aspects of treatment, care and rehabilitation. 1994;3(5):329–38. Epub 1994/10/01. . 7841967 10.1007/BF00451725

[31] Chin HJ, Song YR, Lee JJ, Lee SB, Kim KW, Na KY, et al Moderately decreased renal function negatively affects the health-related quality of life among the elderly Korean population: a population-based study. Nephrol Dial Transplant. 2008;23(9):2810–7. Epub 2008/03/29. 10.1093/ndt/gfn132 . 18372390

[32] Mujais SK, Story K, Brouillette J, Takano T, Soroka S, Franek C, et al Health-related quality of life in CKD Patients: correlates and evolution over time. Clin J Am Soc Nephrol. 2009;4(8):1293–301. Epub 2009/08/01. 10.2215/CJN.05541008 19643926 PMC2723973

[33] Rossi AP, Burris DD, Lucas FL, Crocker GA, Wasserman JC. Effects of a renal rehabilitation exercise program in patients with CKD: a randomized, controlled trial. Clin J Am Soc Nephrol. 2014;9(12):2052–8. Epub 2014/11/22. 10.2215/CJN.11791113 25414318 PMC4255415

[34] Finkelstein FO, Wuerth D, Finkelstein SH. Health related quality of life and the CKD patient: challenges for the nephrology community. Kidney Int. 2009;76(9):946–52. Epub 2009/08/14. 10.1038/ki.2009.307 . 19675529

[35] Finkelstein FO, Story K, Firanek C, Mendelssohn D, Barre P, Takano T, et al Health-related quality of life and hemoglobin levels in chronic kidney disease patients. Clin J Am Soc Nephrol. 2009;4(1):33–8. Epub 2008/11/07. 10.2215/CJN.00630208 18987300 PMC2615698

[36] Sud M, Tangri N, Levin A, Pintilie M, Levey AS, Naimark DM. CKD Stage at Nephrology Referral and Factors Influencing the Risks of ESRD and Death. Am J Kidney Dis. 2014;63(6):928–36. Epub 2014/02/04. 10.1053/j.ajkd.2013.12.008 . 24485146

[37] Wilhelm-Leen ER, Hall YN, KT M, Chertow GM. Frailty and chronic kidney disease: the Third National Health and Nutrition Evaluation Survey. Am J Med. 2009;122(7):664–71 e2. Epub 2009/06/30. 10.1016/j.amjmed.2009.01.026 . 19559169 PMC4117255

[38] Roshanravan B, Khatri M, Robinson-Cohen C, Levin G, Patel KV, de Boer IH, et al A prospective study of frailty in nephrology-referred patients with CKD. Am J Kidney Dis. 2012;60(6):912–21. Epub 2012/07/10. 10.1053/j.ajkd.2012.05.017 22770927 PMC3491110

[39] Lee SJ, Son H, Shin SK. Influence of frailty on health-related quality of life in pre-dialysis patients with chronic kidney disease in Korea: a cross-sectional study. Health and quality of life outcomes. 2015;13:70 Epub 2015/05/30. 10.1186/s12955-015-0270-0 26021987 PMC4460686

[40] Gyamlani G, Basu A, Geraci S, Lee F, Moxey M, Clark M, et al Depression, screening and quality of life in chronic kidney disease. Am J Med Sci. 2011;342(3):186–91. Epub 2011/06/02. 10.1097/MAJ.0b013e3182113d9e . 21629044

[41] Seidel UK, Gronewold J, Volsek M, Todica O, Kribben A, Bruck H, et al Physical, cognitive and emotional factors contributing to quality of life, functional health and participation in community dwelling in chronic kidney disease. PLoS One. 2014;9(3):e91176 Epub 2014/03/13. 10.1371/journal.pone.0091176 24614180 PMC3948783

[42] Campbell KL, Ash S, Bauer JD. The impact of nutrition intervention on quality of life in pre-dialysis chronic kidney disease patients. Clin Nutr. 2008;27(4):537–44. Epub 2008/07/01. 10.1016/j.clnu.2008.05.002 . 18584924

